# Primary care access for mental illness in Australia: Patterns of access to general practice from 2006 to 2016

**DOI:** 10.1371/journal.pone.0198400

**Published:** 2018-06-01

**Authors:** Louise M. Farrer, Jennie Walker, Christopher Harrison, Michelle Banfield

**Affiliations:** 1 Centre for Mental Health Research, The Australian National University, Canberra, Australia; 2 Menzies Centre for Health Policy, University of Sydney, Sydney, New South Wales, Australia; George Institute for Global Health, INDIA

## Abstract

General practice has an important role within the Australian healthcare system to provide access to care and effective management of chronic health conditions. However, people with serious mental illness experience challenges associated with service access. The current paper seeks to examine drivers of access to general practice for people with common and serious mental disorders, compared with people who access care for type II diabetes, a common physical health problem managed in general practice. The Bettering the Evaluation and Care of Health (BEACH) programme provides the most comprehensive and objective measurement of general practitioner activity in Australia. Using BEACH data, this study compared general practice encounters for depression, anxiety, bipolar disorder, schizophrenia, and type II diabetes during a 10-year period between 2006 and 2016. Analysis revealed more frequent encounters for depression compared to anxiety, and a higher representation of women in encounters for bipolar disorder compared to men. The relationship between number of encounters and patient age was strongly associated with the life course and mortality characteristics associated with each disorder. The findings highlight specific challenges associated with access to primary care for people with serious mental illness, and suggest areas of focus to improve the ability of these patients to access and navigate the health system.

## Introduction

The performance of healthcare systems is underpinned by access, that is, the ease with which consumers of healthcare can access services appropriate to their needs [1]. There are many different conceptualisations of access in the health policy literature, and accordingly, measuring access has been described as a ‘complex task’ involving multiple dimensions such as financial and physical accessibility, need, predisposing and enabling factors, availability, and quality [1]. In Australia, general practice has been described as the ‘cornerstone of successful primary health care’ [2] and performs several pivotal roles within the healthcare system, including serving as a first point of contact for medical care, providing ongoing health management, and coordinating care between different health providers [3]. Importantly, general practice performs a gatekeeper role to the rest of the health system; access to most specialist care in Australia requires a referral from a general practitioner (GP) [2]. Thus, in addition to being a first point of contact, general practice has a strong role in facilitating access to other providers in chronic disease management [4].

Mental illness is a major public health concern, accounting for 12% of the total disease burden in Australia, third after cancer and cardiovascular diseases [5]. Anxiety disorders affect 14.4% of the Australian population in any one year, and 5.4% will experience a depressive episode or dysthymia [6]. The 12-month prevalence of bipolar disorder is 1.8% [6], and in 2010, 0.45% of the Australian population were treated for a psychotic illness, most commonly schizophrenia [7]. However, despite their prevalence, service access and effective illness management are major concerns for people with mental health problems. Australians with mental illness report ‘complex and chaotic’ service pathways [8], and insufficiencies in primary care settings, specifically concerning accurate and timely diagnosis, and continuity of care between GPs and specialist mental health service providers [9].

Given the central role of general practice in service access and chronic disease management, it is important to examine how general practice is performing in these areas with regard to mental health problems, compared to other types of problems that are managed in general practice. Proponents of chronic care models such as the World Health Organisation’s Innovative Care for Chronic Conditions framework [10] argue that when examining health system responses to illness management, chronic conditions tend to look more alike than different, both in their impact on patients, families and the system and in how they are managed, compared with acute conditions. This similarity provides an opportunity to compare patterns of access and care across conditions.

The first step in understanding what happens to people with mental health problems in general practice is to examine drivers of access. Previous papers have examined characteristics of general practice access for people with mental health problems at specific points in time [11] (e.g. prior to and immediately following the introduction of the Australian Government’s Better Access to Psychiatrists, Psychologists, and General Practitioners Scheme (Better Access) [12], or have focused solely on depression and/or anxiety disorders [13,14]. There is a gap in the literature regarding access for people with low prevalence mental illnesses (e.g. schizophrenia and bipolar disorder). There is also a current lack of investigation into how general practice access for mental health problems compares to access for physical health problems.

The current paper seeks to fill these gaps by comparing general practice encounters for serious mental illnesses (schizophrenia and bipolar disorder), common mental disorders (depression and anxiety) and Type II diabetes (T2D). T2D is commonly used as an archetype for chronic illness in major frameworks [15]. This is unsurprising, given that diabetes has been recognised as the world’s fastest growing chronic illness [16]. Diabetes is a major and rising public health concern in Australia, affecting 7–8% of men and 5–7% of women [17,18].

The Bettering the Evaluation and Care of Health (BEACH) programme provides the most comprehensive and objective measurement of GP activity in Australia [19,20]. Using BEACH data, this paper examines 10 years of general practice encounters for depression, anxiety, bipolar disorder, schizophrenia, and T2D to explore patterns of access to primary care.

## Methods

The BEACH programme was a continuous, representative, national cross-sectional programme, which was conducted over 18 years, from April 1998 to June 2016. An ever changing, random sample of 1000 active GPs was surveyed each year, with each providing details on 100 consecutive patient encounters. The database includes approximately 1.78 million patient encounter records by approximately 11,000 GPs. Encounter information recorded by GPs included reason for visit, problem managed, treatment delivered, and referral/s to other health professionals. In addition, GPs recorded up to three Medicare Benefits Schedule (MBS) item numbers per encounter. The Medicare Benefits Schedule is a listing of the services provided by the Australian Government under Medicare. For detailed study methods of the BEACH programme, see [19,20].

The ethical aspects of the BEACH study were approved by the Human Research Ethics Committee of the University of Sydney (protocol 2012/130). Informed consent was obtained from each patient in order for the GP to record their encounter details. Patients were provided with an information card, and asked for their permission for their data to be included in the BEACH programme. The data collected by GPs was not sufficient to identify an individual patient. Extraction and analysis of BEACH data for the current study was approved by The Australian National University Human Research Ethics Committee (protocol 2017/344).

### Participants

GPs reported up to four problems managed per encounter in a free text field, and during data entry, trained research staff coded each problem managed according to the International Classification of Primary Care Version 2 PLUS (ICPC-2 PLUS) [21]. These data were then automatically secondarily classified to ICPC-2 [22]. For the current analyses, patients who had T2D (ICPC-2 code ‘T90’), anxiety (‘P01’,‘P74’), depression (‘P76’), bipolar disorder (‘P73’), and/or schizophrenia (‘P72’) managed at the encounter by GPs were analysed. The code ‘P01’ above refers to ‘feeling anxious’, and the code ‘P73’ refers to affective psychosis, which is considered to be equivalent to bipolar disorder.

### Measures

Patient characteristics extracted for the current analyses were patient sex, age group, whether the patient was a holder of a government health care card, whether the patient identified as Aboriginal or Torres Strait Islander, and patient postcode. Postcode was used to derive a patient’s Australian State or Territory of residence, and their geographical location as per the Australian Statistical Geography Standard (ASGS). The ASGS provides a framework to define geographical concepts such as urban or rural [23]. Five ASGS-defined areas were used in the current study: major cities, inner regional, outer regional, remote, and very remote.

### Analysis

Data for the current study were analysed using SAS software (version 9.4) [24]. Percentages and confidence intervals were used to describe the distribution of encounters, problems managed, and patient characteristics. A significant difference between group percentages was determined by non-overlapping 95% confidence intervals. This method is a conservative approach to assessing significant differences between groups [25]. In the overall BEACH dataset, the representativeness of the participating GPs was assessed annually by comparing the sample with the larger population from which the GPs were drawn. Where there was an over- or under-representation in a particular sex or age group, age-sex weights were applied. Similarly, weights were applied to account for variation in the numbers of services provided by GPs in a given year. Due to the cluster sample design of the BEACH programme, adjustments were made to confidence intervals to account for the correlation between observations within clusters [20].

## Results

### Total encounters and 10-year trends

A total of 9,721 GPs took part in the BEACH project from 2006–2016, and 972,100 patient encounters were recorded during this period. Overall, depression was managed at 4.5% of these encounters (n = 43,616), T2D was managed at 3.6% (n = 34,668), anxiety was managed at 2.1% (n = 20,657), schizophrenia was managed at 0.5% (n = 4,534), and bipolar disorder was managed at 0.3% of these encounters (n = 2,919).

**Fig 1:**
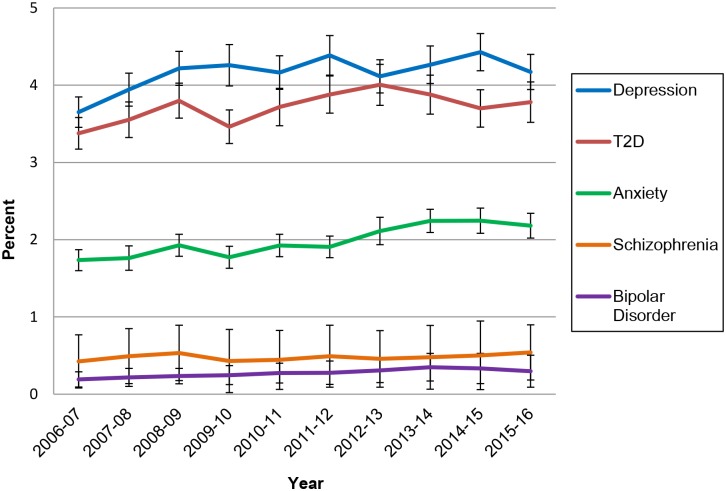
The percentage (and 95% confidence interval) of GP encounters by problem managed and year (April 2006 to March 2016).

Fig 1 presents the percentage of total encounters in which each of the five problems were managed by year between 2006 and 2016. The percentage of GP encounters for T2D increased from 3.4% of total encounters in 2006–07 to 4.0% in 2012–13 and remained stable for the following three years. Likewise, the percentage of GP encounters for depression increased from 3.7% of total encounters in 2006–07 to 4.2% in 2008–09, where it remained stable. The percentage of encounters where anxiety was managed also increased significantly across the study period. The proportion of GP encounters for the management of bipolar disorder or schizophrenia remained steady across the 10 year period from 2006 to 2016.

### Management of multiple conditions

Patients may have had more than one of our selected problems managed during a single consultation. Table 1 displays the percentage of encounters where more than one problem of interest was managed. As less than 10% of encounters had multiple problems of interest managed, an adjustment for overlap was not conducted [26]. Of encounters involving schizophrenia, 6.4% also had diabetes managed. This was significantly greater than for encounters involving depression, anxiety, or bipolar disorder.

**Table 1:** Percentage (and 95% confidence intervals) of encounters with more than one problem managed (April 2006–March 2016).

	T2D (*n* = 34668)	Anxiety (*n* = 20657)	Depression (*n* = 43616)	Bipolar Disorder *(n* = 2919)	Schizophrenia (*n* = 4534)
	*%* (95% CI)	*%* (95% CI)	*%* (95% CI)	*%* (95% CI)	*%* (95% CI)
T2D	-	1.7 (1.6–1.9)	2.9 (2.7–3.0)	3.9 (3.2–4.7)	6.4 (5.7–7.2)
Anxiety	1.0 (0.9–1.1)	-	1.3 (1.1–1.4)	2.0 (1.5–2.5)	1.9 (1.5–2.4)
Depression	3.6 (3.4–3.8)	2.7 (2.4–3.0)	-	1.6 (1.2–2.1)	3.5 (2.9–4.0)
Bipolar disorder	0.3 (0.3–0.4)	0.3 (0.2–0.4)	0.1 (0.1–0.1)	-	0.3 (0.1–0.4)
Schizophrenia	0.8 (0.7–0.9)	0.4 (0.3–0.5)	0.4 (0.3–0.4)	0.4 (0.2–0.7)	-

### Encounters by patient characteristics

**Fig 2:**
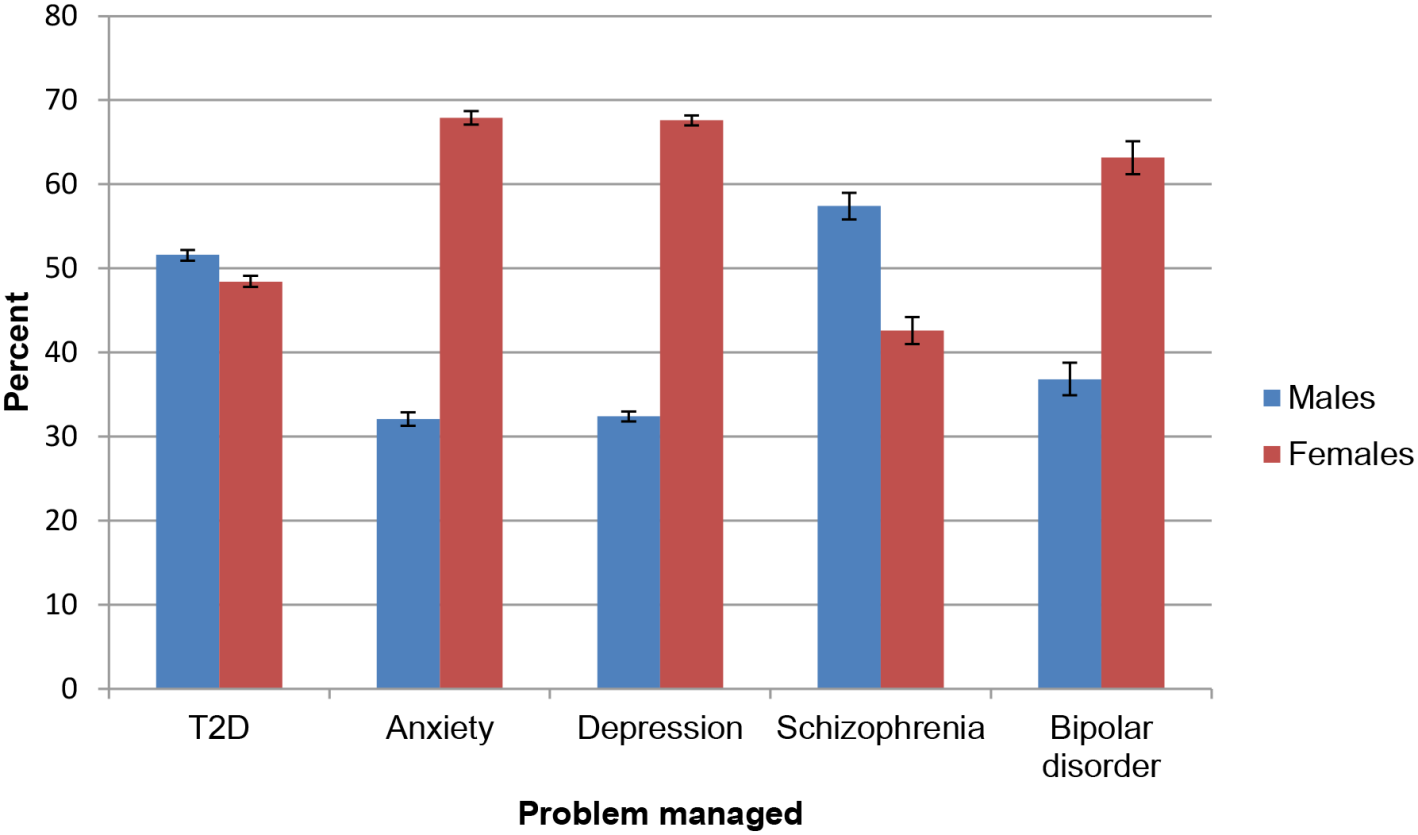
Percentage and 95% confidence intervals of encounters by sex and problem managed (April 2006–March 2016).

S1 Table summarises encounters by patient characteristics, for each of the disorders examined. Females were represented in a significantly higher proportion of encounters for T2D, anxiety, depression, and bipolar disorder, whereas a significantly higher proportion of males were represented in encounters for schizophrenia (Fig 2).

The pattern of distribution of encounters by age group was similar for the four mental disorders compared with T2D, but there were some significant differences between the mental disorders. Compared to depression, anxiety and bipolar disorder, significantly fewer encounters for schizophrenia were with those aged less than 24. Approximately a third of encounters for depression and anxiety, and approximately 40% of encounters for bipolar disorder and schizophrenia were with those aged between 25 and 44 years. A significantly greater proportion of patients who were having schizophrenia managed were aged 45–64 years than other disorders. Over 90% of encounters for T2D were with patients aged over 45 years, with significantly higher proportions of encounters with people aged over 65 than any of the four mental disorders (2–3 times higher).

Within each Australian State or Territory, the proportion of problems managed was similar except for schizophrenia. In Western Australia, a significantly lower proportion of encounters involved schizophrenia. Patterns were also similar for location by ASGS, although a significantly greater proportion of encounters for with bipolar disorder were in inner regional areas. There was a trend towards higher proportions of encounters for anxiety and schizophrenia in major cities.

Compared to all other problems managed, a significantly greater proportion of encounters for schizophrenia were with patients holding a health care card (88.3%). The percentage of encounters with patients holding health care cards was similar for bipolar disorder and T2D, but significantly higher than for encounters for anxiety and depression (around half with a health care card).

People of Aboriginal and Torres Strait Islander descent were the more highly represented in encounters for schizophrenia and T2D, compared to encounters for anxiety, depression, and bipolar disorder.

## Discussion

This paper provides a comprehensive picture of the rates of primary care access for high and low prevalence mental disorders in Australia from 2006–2016. This paper also examines the characteristics of those who accessed general practice care for these disorders compared to those who accessed care for T2D, a comparable chronic physical health condition commonly managed in general practice. Among the five health conditions examined, depression was the most common disorder to present in general practice (approximately 1 in 20 encounters), followed by T2D, anxiety disorders, schizophrenia, and bipolar disorder. Depression was managed at twice the rate that anxiety was managed, which is surprising given that Australians are two to three times more likely to experience an anxiety disorder than depression or dysthymia [6]. Previous studies, however, have found that people more commonly present to general practice for depression than anxiety [11,13,27]. There is some evidence that the public views general practitioners as more helpful for depression than other health professionals [28,29]. Help-seeking also relies on the ability to recognise the symptoms of a mental health problem as being ‘treatment-worthy’; a concept known as mental health literacy [30]. Studies of mental health literacy have found higher levels of depression literacy than anxiety literacy in the community [31], which may be due to several factors, including a greater emphasis on depression in awareness campaigns, and a tendency to normalise the experience of anxiety symptoms as everyday ‘stress’ [32]. Encounters involving bipolar disorder in general practice were lower than population estimates of prevalence, which may be due to several factors, including failure by GPs to accurately recognise or diagnose bipolar disorder, or low attendance of patients with bipolar disorder to general practice [33]. Rates of access to general practice for schizophrenia were commensurate with population estimates of prevalence. This is consistent with studies demonstrating that most people diagnosed with schizophrenia have regular contact with a general practitioner [7,34], often for administration of antipsychotic medication and/or the management of physical health problems that commonly co-occur with psychotic illnesses [35]. Compared to those who access public mental health services, people with schizophrenia who access general practice experience fewer symptoms, improved functioning, and lower service use [36].

As has been noted previously (e.g. [11]), BEACH data provide an opportunity to examine the impact of government initiatives such as the Better Access scheme [12]. In the current study, a significant increase in the number of encounters associated with depression was observed during the 2 to 3 years following the introduction of Better Access (i.e. 2007–8 and 2008–9). The Better Access initiative was designed to improve treatment rates for common mental disorders [37,38], which may partially explain why no changes were observed in general practice access rates for schizophrenia and bipolar disorder in the years following the introduction of Better Access.

Schizophrenia was the most likely of the mental disorders examined to be co-morbid with T2D in general practice encounters. This is consistent with a recent meta-analysis reporting that the prevalence of diabetes is significantly higher in people with serious mental illness [39]. This may be due to several factors including a link between metabolic syndrome and the use of antipsychotic medications [40], the vulnerability of people with schizophrenia to risk factors such as smoking, high alcohol consumption, poor diet, and lack of exercise [41], or a genetic predisposition [42,43]. High co-morbidity with physical health problems presents a challenge for the treatment of severe mental illnesses, which further highlights the important role of GPs to detect co-morbidity and provide or coordinate appropriate treatment.

Greater numbers of women than men had depression and anxiety managed at general practice encounters, which mirrors an established finding in the literature that women experience these disorders at higher rates than men [44,45]. Numbers of men and women presenting to general practice for T2D and schizophrenia were also consistent with sex distributions in the population prevalence of these disorders [7,17,18]. Interestingly, a significantly higher number of females presented to general practice for bipolar disorder than males, despite equal numbers of men and women experiencing bipolar disorder in the general population [46]. This suggests that the sex distribution of disease prevalence is only one potential driver of access to treatment. Sex has also been shown to affect symptom expression, illness course, and patterns of co-morbidity in mental disorders generally, which, alone or in combination, may also influence treatment seeking behaviour [47]. It is possible that patterns of physical health comorbidities in women with bipolar disorder result in an increased likelihood that they will present to general practice [46,48].

Age distributions in general practice encounters were consistent with the life course of the disorders examined. The majority of encounters for T2D were among patients aged 45 years and older, which is unsurprising given that adults of this age are most likely to experience risk factors and symptoms associated with T2D [49]. There were relatively few encounters for depression, anxiety, schizophrenia, and bipolar disorder among people aged younger than 24 years, which may reflect several issues including a common age of onset during early adulthood (for schizophrenia in particular), significant delays between onset of symptoms and accurate diagnosis, ambivalence about help-seeking, or, in the case of serious mental illnesses, an inability for professionals to diagnose these disorders according to current diagnostic guidelines. There is also evidence of increased mortality associated with severe mental illnesses such as schizophrenia and bipolar disorder [50,51]. This is reflected in the sharp decline observed in encounters associated with these disorders among patients aged 65 or older.

People presenting to general practice with schizophrenia were more likely to hold a government-issued health care card than people with other disorders. Health care cards are designed to provide those who face socioeconomic disadvantage with reductions in costs associated with health care. People with severe mental illnesses face individual, community-level, and system-level barriers to workforce participation [52], and thus, may be at greater risk of socioeconomic disadvantage.

Aboriginal and Torres Strait Islanders were more highly represented in general practice encounters for T2D and schizophrenia, compared to other disorders. People from these backgrounds are three times more likely to have diabetes than the general Australian population [53]. Increased risk of psychosis among Aboriginal and Torres Strait Islander peoples [54] has been partially attributed to high rates of substance misuse [55]. Western conceptualisations of illness tend to focus on modifiable risk factors such as diet and exercise (in the case of T2D) or genetic and biological markers (in the case of schizophrenia). However, effective detection and management of diabetes and schizophrenia in Aboriginal and Torres Strait Islander populations requires a recognition of the historical, cultural, and societal factors that underpin the development of these disorders, including the role of spirituality, education, employment, income, housing, access to services, connection with land, racism, and incarceration [56,57].

People with bipolar disorder were more highly represented in inner regional areas, whereas a higher proportion of people with schizophrenia were located in major cities. Higher prevalence of schizophrenia in urban environments is a well-documented finding, given the tendency for people with psychosis to live in areas with greater access to inexpensive accommodation, formal and informal services, and anonymity [58]. Other studies have found no significant differences in the prevalence of mental health problems in urban and rural areas [59,60], however, these studies have largely focused on high prevalence disorders like depression, anxiety, and substance use disorders. No studies in Australia have examined urban and rural differences in bipolar disorder specifically, but rurality has been associated with inadequacies in access to high quality services [61], which is important when considering how to optimise the management of serious mental illnesses.

### Limitations

The main limitation of this study is that the BEACH dataset only records problems that were actively managed by GPs during each encounter. For example, while more than one problem could be recorded per encounter, it is likely that these records are an underestimate of comorbidity, as patients may have had more conditions than those managed at an encounter. As a cross-sectional study, the results also only reflect trends over time, but do not allow for longitudinal analyses of individual patients. This would likely have resulted in the inclusion of patients with depression and anxiety with a discrete episode (i.e., not ongoing) rather than chronic disorder. Moreover, access is commonly considered to reflect the first contact that a patient makes with a service for a given problem. As rates of encounters were used as a proxy measure of access in the current study, this may have resulted in an overrepresentation of particular conditions relative to their population prevalence, where there were higher rates of repeat contacts (e.g. people with schizophrenia presenting to general practice for regular administration of depot injections). Anxiety was recorded using two codes, one of which refers to a diagnosis of an anxiety disorder (P74), and another (P01) which refers to the experience of symptoms of anxiety. The use of two codes to record anxiety may have resulted in an overestimation of the prevalence of anxiety in the current study.

## Conclusion

This study aimed to investigate patterns of access to general practice for people with common mental disorders, serious mental illness and chronic physical illness. As the primary point of access to our healthcare system, a crucial step in optimising care for people with mental health problems involves understanding whether access is commensurate with the community prevalence, and age and sex distributions of these disorders. Similarly, it is important to examine other factors that may affect access, including location, cultural background, and socioeconomic characteristics.

The patterns of encounters for T2D were broadly consistent with population prevalence, life course and patient characteristics, and the encounters over time reflected the increasing prevalence of the disease. However, whereas access was commensurate with regard to some of the factors examined for mental health problems, the current paper highlights some important areas of concern. For three of the four mental health conditions, men accessed care significantly less than women: there is a need to understand and address the factors that underlie lower rates of men accessing general practice, particularly for disorders where no sex differences exist in the population (e.g. bipolar disorder). Also, whilst the data suggest adequate utilisation of general practice by patients with schizophrenia in Australia, which presents good opportunity for optimal management of the mental and physical health concerns of these patients, it is concerning that people with bipolar disorder, who may face similar concerns, have the lowest rates of access overall. Similarly, the relatively low rate of access for anxiety disorders is particularly troubling given the prevalence of these disorders in the community. The results also suggest that socio-demographic factors such as location of residence, socio-economic disadvantage, and cultural background influence the presentation of serious mental illness in general practice, and this knowledge may be used to highlight and modify how health care resources is distributed geographically and provided to patients.

The results have some important implications for service planning and policy. Whilst access by people with a physical health condition followed expected patterns, barriers exist for some groups with mental health problems. Further, the frequently-used grouping of mental disorders by prevalence may mask the under-utilisation of services for both low and high prevalence disorders. In some cases, this may reflect a perception (or reality) that general practice is not the most appropriate avenue for treatment, but given the centrality of general practice for access to the broader Australian health system, further work is needed to understand how people with chronic mental health problems can be encouraged to access this important component of care.

## Supporting information

S1 Table Patient characteristics by problem encounters (April 2006–March 2016). (DOCX)
