# Supplementary material for: Primary care access for mental illness in Australia: Patterns of access to general practice from 2006 to 2016
Source: PLoS One. 2018 Jun 1;13(6):e0198400. doi: 10.1371/journal.pone.0198400 (PMC5983527; doi:10.1371/journal.pone.0198400)
Supplement: S1 Table — (DOCX) [file pone.0198400.s001.docx]

**Supporting information**

**S1 Table**. Patient characteristics by problem encounters (April 2006 – March 2016)

|  | Type 2 Diabetes  *n =* 34,668 | Anxiety  *n =* 20,657 | Depression  *n =* 43,616 | Bipolar disorder  *n =* 2,919 | Schizophrenia  *n =* 4,534 |
| --- | --- | --- | --- | --- | --- |
|  | **% (95% CI)** | **% (95% CI)** | **% (95% CI)** | **% (95% CI)** | **% (95% CI)** |
| Sex |  |  |  |  |  |
| Male | 51.6 (50.9-52.2) | 32.1 (31.3-32.9) | 32.4 (31.8-33) | 36.8 (34.9-38.8) | 57.4 (55.8-59) |
| Female | 48.4 (47.8-49.1) | 67.9 (67.1-68.7) | 67.6 (67.0-68.2) | 63.2 (61.2-65.1) | 42.6 (41.0-44.2) |
| *Missing* | *n =* 309 | *n =* 160 | *n* = 380 | *n =* 26 | *n =* 49 |
|  |  |  |  |  |  |
| Age group |  |  |  |  |  |
| 5-14 years | 0.1 (0.0-0.1) | 2.9 (2.6-3.1) | 0.6 (0.5-0.7) | 0.2 (0.1-0.4) | 0.1 (0.0-0.2) |
| 15-24 years | 0.4 (0.3-0.5) | 9.1 (8.6-9.6) | 9.4 (9.1-9.8) | 8.1 (7.0-9.2) | 4.1 (3.5-4.7) |
| 25-44 years | 6.4 (6.1-6.7) | 32.3 (31.6-33.1) | 33.2 (32.7-33.8) | 40.4 (38.5-42.3) | 38.7 (37.1-40.3) |
| 45-64 years | 37.3 (36.7-37.9) | 32.2 (31.5-32.8) | 37.4 (36.9-37.9) | 36.7 (34.8-38.5) | 42.1 (40.5-43.7) |
| 65-74 years | 29.2 (28.7-29.7) | 11.3 (10.8-11.8) | 10.0 (9.7-10.4) | 8.8 (7.7-9.9) | 8.8 (7.9-9.8) |
| 75+ years | 26.6 (26-27.2) | 12.0 (11.5-12.5) | 9.3 (8.9-9.7) | 5.8 (4.8-6.7) | 6.0 (5.3-6.8) |
| *Missing* | *n =* 269 | *n =* 165 | *n* = 372 | *n =* 23 | *n =* 43 |
|  |  |  |  |  |  |
| Health care card | 68.8 (68.1-69.5) | 51.5 (50.5-52.5) | 49.2 (48.5-49.9) | 68.3 (66.4-70.2) | 88.3 (87.2-89.4) |
| *Missing* | *n =* 2074 | *n =* 1576 | *n* = 3360 | *n =* 172 | *n =* 236 |
|  |  |  |  |  |  |
| Aboriginal/Torres Strait Islander | 3.6 (3.1-4.2) | 1.6 (1.3-1.8) | 1.8 (1.6-2.0) | 2.1 (1.4-2.7) | 3.9 (3.0-4.8) |
| *Missing* | *n =* 2458 | *n =* 1809 | *n* = 4049 | *n =* 203 | *n =* 260 |
|  |  |  |  |  |  |
| State |  |  |  |  |  |
| ACT | 1.3 (1.0-1.5) | 1.8 (1.4-2.2) | 1.8 (1.5-2.2) | 2.4 (1.6-3.2) | 1.7 (1.1-2.3) |
| NSW | 35.4 (34.1-36.7) | 32.0 (30.6-33.3) | 32.4 (31.2-33.6) | 32.9 (30.5-35.2) | 35.6 (33.2-37.9) |
| VIC | 23.8 (22.7-25.0) | 27.5 (26.2-28.9) | 25.4 (24.3-26.5) | 25.9 (23.7-28.0) | 29.3 (26.9-31.7) |
| QLD | 17.6 (16.6-18.6) | 17.8 (16.7-18.9) | 18.7 (17.8-19.7) | 17.2 (15.3-19.0) | 14.6 (13.0-16.2) |
| SA | 8.7 (7.9-9.5) | 7.8 (7.0-8.6) | 8.1 (7.4-8.8) | 9.4 (8.0-10.9) | 10.6 (8.8-12.4) |
| WA | 9.4 (8.6-10.2) | 9.1 (8.2-9.9) | 9.6 (8.9-10.3) | 8.2 (6.9-9.5) | 5.6 (4.7-6.5) |
| TAS | 3.1 (2.7-3.6) | 3.5 (2.9-4.0) | 3.3 (2.8-3.7) | 3.6 (2.6-4.6) | 2.4 (1.8-3.0) |
| NT | 0.8 (0.5-1.0) | 0.6 (0.4-0.8) | 0.7 (0.5-0.9) | 0.5 (0.1-0.8) | 0.3 (0.1-0.5) |
| *Missing* | *n =* 732 | *n =* 397 | *n* = 919 | *n =* 58 | *n =* 90 |
|  |  |  |  |  |  |
| ASGS |  |  |  |  |  |
| Major Cities | 65.4 (64.2-66.7) | 72.5 (71.2-73.7) | 67.8 (66.6-68.9) | 63.7 (61.4-66.1) | 70.6 (68.4-72.8) |
| Inner Regional | 21.9 (20.8-23.0) | 19.5 (18.4-20.6) | 22.1 (21.1-23.1) | 25.5 (23.4-27.7) | 20.6 (18.8-22.5) |
| Outer Regional | 10.5 (9.6-11.3) | 7.2 (6.6-7.9) | 9.0 (8.4-9.7) | 9.2 (7.8-10.7) | 7.7 (6.3-9.1) |
| Remote | 1.5 (1.1-1.9) | 0.6 (0.4-0.8) | 0.9 (0.7-1.0) | 1.0 (0.6-1.4) | 0.8 (0.5-1.1) |
| Very Remote | 0.7 (0.4-1.0) | 0.2 (0.1-0.2) | 0.3 (0.2-0.4) | 0.5 (0.2-0.8) | 0.3 (0.1-0.4) |
| *Missing* | *n =* 764 | *n =* 422 | *n* = 962 | *n =* 60 | *n =* 95 |

Note. Ages 0-4 years were removed due to low numbers
